# Transgenic pigs to the rescue

**DOI:** 10.7554/eLife.37641

**Published:** 2018-05-22

**Authors:** Björn Petersen

**Affiliations:** Department of BiotechnologyFriedrich-Loeffler-InstitutGreifswaldGermany

**Keywords:** transgenic pigs, Suidae, nitrogen, phosphorus, manure, pollution, Other

## Abstract

Genetically engineered pigs that digest their food better could help to reduce phosphorus and nitrogen pollution.

**Related research article** Zhang X, Li Z, Yang H, Liu D, Cai G, Li G, Mo J, Wang D, Zhong C, Wang H, Sun Y, Shi J, Zheng E, Meng F, Zhang M, He X, Zhou R, Zhang J, Huang M, Zhang R, Li N, Fan M, Yang J, Wu Z. 2018. Novel transgenic pigs with enhanced growth and reduced environmental impact. *eLife* **7**:e34286. doi: 10.7554/eLife.34286

In the United States alone, two nutrients, nitrogen and phosphorus, pollute the water in more than 100,000 miles of streams and rivers and almost 2.5 million acres of lakes, reservoirs and ponds (EPA, 2018). Swine manure is one of the main factors responsible for this large-scale contamination. A by-product of the pig farming industry, this bodily waste is rich in both nitrogen and phosphorus, and is widely used as fertilizer. Now, in eLife, Zhenfang Wu and colleagues at the South China Agricultural University and other institutes in China, Canada, and the United States – including Xianwei Zhang and Zicong Li as joint first authors – report that genetically engineered pigs which release less of these nutrients could be a solution to the problem (Zhang et al., 2018).

Nitrogen and phosphorus naturally occur in aquatic ecosystems, where they support the growth of algae and aquatic plants. But when large quantities of these nutrients enter the environment – especially streams, rivers, bays and coastal waters – they can boost the growth of green and blue algae. These algal blooms drain the oxygen from the water, ultimately asphyxiating aquatic life. Some algal blooms also produce toxins and support bacterial growth that can be harmful to people and animals in contact with the contaminated water.

Nitrogen and phosphorus pollution can also affect human health. Nitrate and nitrite, which derive from nitrogen, often seep into groundwater in rural areas and can be damaging to children and pregnant women if they end up in drinking water (Cockburn et al., 2013; Richard et al., 2014). Nitrates prevent the blood from efficiently carrying oxygen to the organs, and this can cause deadly methemoglobinemia, or ‘blue baby’ disease, in infants.

Reducing the levels of nitrogen and phosphorus in swine manure is one way to control this pollution. Pigs excrete large amounts of these chemicals, partly because they cannot digest phytates (which are used by plants to store phosphorus) or non-starch polysaccharides, two types of molecules that are present in their feedstuff. This means that up to 70% of the phosphorus given to a grown pig will be excreted as bodily waste (Dourmad et al., 1999). It is also estimated that a single boar can produce almost 18kg of nitrogen each year (DEFRA, 2017). Moreover, the fact that pigs cannot digest phytates or non-starch polysaccharides prevents them from accessing many of the nutrients in their feed, which limits their energy intake.

Almost two decades ago, researchers used genetic techniques to engineer a transgenic ‘Enviropig’ that could process phytates (Golovan et al., 2001). Now Zhang et al. have created transgenic pigs that express enzymes which allow them to digest both phytates and non-starch polysaccharides. Zhang et al. took five genes from bacteria and fungi and introduced them into the genomes of pigs to create animals that expressed four bacterial enzymes (two types of β-glucanase, xylanase, phytase) in their salivary glands. In the mouth of the animals, the enzymes could break phytates and non-starch polysaccharides into molecules that the pigs could then digest. The modified animals produced bodily waste that contained up to 24% less nitrogen and 44% less phosphorus compared with other pigs on the same diet. The results were slightly lower than those previously reported for transgenic pigs that can just break down phytates, possibly because of differences in the expression levels of the transgenes and changes in diet (Golovan et al., 2001; Forsberg et al., 2013; Meidinger et al., 2013).

Besides excreting fewer polluting nutrients, the transgenic pigs also grew better and fattened up more quickly. In fact, on average, they put on 24% more weight every day than their non-modified counterparts. As a result, they could be slaughtered nearly a month earlier. This is an advantage that ‘Enviropig’ did not have.

By growing fast, requiring less food and producing fewer damaging chemicals, the pigs developed by Zhang et al. might create a win-win situation for both farmers and environment.
